# Geographical Analysis of the Distribution and Spread of Human Rabies in China from 2005 to 2011

**DOI:** 10.1371/journal.pone.0072352

**Published:** 2013-08-26

**Authors:** Danhuai Guo, Hang Zhou, Yan Zou, Wenwu Yin, Hongjie Yu, Yali Si, Jianhui Li, Yuanchun Zhou, Xiaoyan Zhou, Ricardo J. Soares. Magalhães

**Affiliations:** 1 Scientific Data Center, Computer Network Information Center, Chinese Academy of Sciences, Beijing, China; 2 Key Laboratory of Surveillance and Early-warning on Infectious Disease, Division of Infectious Disease, Chinese Center for Disease Control and Prevention, Beijing, China; 3 Department of Female Clinical Research, National Research Institute for Family Planning, Beijing, China; 4 Ministry of Education Key Laboratory for Earth System Modeling, Center for Earth System Science, Tsinghua University, Beijing, China; 5 Emergency Centre for the Control of Transboundary Animal Diseases, Food and Agriculture Organization of the United Nations, Beijing, China; 6 University of Queensland, Infectious Disease Epidemiology Unit, School of Population Health, Herston, Queensland, Australia; Thomas Jefferson University, United States of America

## Abstract

**Background:**

Rabies is a significant public health problem in China in that it records the second highest case incidence globally. Surveillance data on canine rabies in China is lacking and human rabies notifications can be a useful indicator of areas where animal and human rabies control could be integrated. Previous spatial epidemiological studies lacked adequate spatial resolution to inform targeted rabies control decisions. We aimed to describe the spatiotemporal distribution of human rabies and model its geographical spread to provide an evidence base to inform future integrated rabies control strategies in China.

**Methods:**

We geo-referenced a total of 17,760 human rabies cases of China from 2005 to 2011. In our spatial analyses we used Gaussian kernel density analysis, average nearest neighbor distance, Spatial Temporal Density-Based Spatial Clustering of Applications with Noise and developed a model of rabies spatiotemporal spread.

**Findings:**

Human rabies cases increased from 2005 to 2007 and decreased during 2008 to 2011 companying change of the spatial distribution. The ANN distance among human rabies cases increased between 2005 and 2011, and the degree of clustering of human rabies cases decreased during that period. A total 480 clusters were detected by ST-DBSCAN, 89.4% clusters initiated before 2007. Most of clusters were mainly found in South of China. The number and duration of cluster decreased significantly after 2008. Areas with the highest density of human rabies cases varied spatially each year and in some areas remained with high outbreak density for several years. Though few places have recovered from human rabies, most of affected places are still suffering from the disease.

**Conclusion:**

Human rabies in mainland China is geographically clustered and its spatial extent changed during 2005 to 2011. The results provide a scientific basis for public health authorities in China to improve human rabies control and prevention program.

## Introduction

Rabies is a widely distributed zoonotic infectious disease and worldwide about 55,000 deaths are estimated to occur each year [1], [2]. China is the second after India in the annual incidence of human rabies cases. From 1950 to 2011 China experienced several serious human rabies epidemics [3], [4]. While over the past fifty years the annual number of cases has decreased, the epidemic situation remains serious in that a total of 1,917 cases were reported in 2011. Unsuccessful control of canine rabies and inadequate post-exposure prophylaxis (PEP) of patients are thought to be the main factors leading to the high incidence of human rabies in China [5], [6]. Notably each year rabies infections appeared in areas without previous history of infection.

Numerous studies have been conducted to investigate the epidemiology and transmission dynamics of human rabies in China across different temporal and geographical scales [5]–[11]. Phylogenic analysis of Chinese rabies viruses from 1969 and 2009 demonstrated that infection had been transmitted intra-provincially and extra-provincially due to human-related activities [7]. Time-series analysis of human rabies in China has shown seasonal trends in infection in that the number of cases in summer and autumn was higher compared to that in spring and winter [11].

Understanding the spatial distribution of rabies in animals or humans and its transmission dynamics are critical for forecasting its emergence and spread into new geographic regions, and help inform targeted interventions. Unfortunately, limited data are currently available about the burden of canine rabies in China given the poor surveillance of animal rabies in the country [12], [13]. As human rabies is a dead end infection, reported human rabies cases are a useful indicator for public health authorities to plan and implement strategies for the control of human rabies transmitted by dogs such as, promote community awareness and knowledge of dog bite prevention, first aid and management of animal bites, PEP and responsible dog ownership [14]. Studies investigating the spatial patterns of rabies in humans and animals allow the identification of areas where animal-to-human transmission is at its highest. Previous studies have shown significant local spatial variation of rabies infections in raccoons and skunks [15]–[17]. More recently, a model has been developed to forecast raccoon rabies emergence using surveillance data [18]. The only spatial epidemiological study of canine rabies has shown that the spatial and temporal distribution of canine rabies was not evenly distributed [19]. Meanwhile, some studies have investigated the geographical distribution and spread of human rabies [20]–[22]. In China, spatial analyses of human rabies cases have shown that while the number of human rabies cases decreased from 2007 to 2011 the number of counties with human rabies cases reported did not change significantly [10]. More recently, analysis of human rabies from 2004 to 2010 in Henan Province demonstrated that the distribution of human rabies was spatially clustered at the county level [23].

To accurately identify human rabies spatiotemporal distribution and spread patterns, it is necessary to analyze human rabies data at the case level. However, all the previous spatial epidemiological investigations were based on aggregated areas, i.e. at the level of administrative districts such as province/state, county or township. The use of analyses based on spatially aggregated data are likely to miss important spatial patterns of disease distribution in that transmission of rabies is not restricted by administrative boundaries and its occurrence is determined by the surrounding geographic environment, economic condition and human behavior which are spatially heterogeneous [5]. Kernel density mapping allows the visualization of point-based disease events and helps to identify areas where outbreak intensity is highest and generates a testable hypothesis about the presence of disease clusters. Spatiotemporal methods for the detection of disease clusters help confirm the hypothesis and provide clues to the causes of the disease process, and assist to improve control and prevention program [24].

In this study we aim to quantify the spatiotemporal distribution of human rabies cases in China from 2005 to 2011, detect spatiotemporal clusters of human rabies and model the transmission trend of rabies in the country. The objective is to provide a scientific basis for improved targeted human rabies control interventions in China.

## Materials and Methods

### Ethics Statement

The authors were provided individual-level, anonymised data which were then stored in a password-encrypted file in a single personal computer. The authors are not authorized by the data providers to disseminate the data used in the analysis nor to generate copies of the original data file.

### Rabies Data

Data used in this study were abstracted from the National Notifiable Disease Reporting System (NDRS), the information system of infectious diseases of mandatory notification in mainland China. Human rabies is one of the Class B notifiable diseases according to the Chinese Law on Prevention and Treatment of Infectious Diseases, and covered by NDRS. Each human rabies case includes information regarding patient ID, sex, age, occupation, residential address, 8-digit administrative code, date of infection and address of reporting hospital. The residential address is detailed to street level with the house number if the patient lives in urban areas and to village level if he/she lives in rural areas.

From Jan 1, 2005 to Dec 31, 2011, 29 provincial districts reported human rabies cases and 17,760 cases were reported. For the other 5 districts, no cases were reported in Qinghai and Xizang and no data were available in Hong Kong, Macau and Taiwan. Only sporadic cases were reported in Gansu, Heilongjiang, Jilin, Liaoning, Ningxia and Xinjiang. The continuously infected provincial districts included Anhui, Beijing, Chongqing, Fujian, Guangdong, Guangxi, Guizhou, Hainan, Hebei, Henan, Hubei, Hunan, Inner Mongolia, Jiangsu, Jiangxi, Shaanxi, Shandong, Shanghai, Shanxi, Sichuan, Tianjin, Yunnan and Zhejiang. The infection was mainly concentrated in the Southeastern part of China, covering approximately 46.98% of the nation’s land, 83.87% of the nation’s total population and 88.52% of Gross Domestic Product (http://www.stats.gov.cn/).

### Geocoding and Projection

The residential address was geocoded and the household was designated the geographical unit of analysis. The address would be processed to a complete and normalized address with five or six hierarchical administrative district names; then to match with gazetteer records. If this step failed, as ambiguity and uncertainty naturally occur in address inputting, the Google® geocoding service, which provides users an optional interface by integrating with vector map satellites, would be called to match the unmatched addresses. If all the previous steps failed, the location of townships recorded as 8-digit administrative code would be used. For convenience of further spatial analysis, we chose WGS_84_UTM_49N (http://spatialreference.org/ref/epsg/32649/) as the unified projected coordinate system, because its central meridian (111 degree) was located near the center of our study area.

### Gaussian Kernel Density Surface Analysis

Gaussian kernel density analysis produces a continuous density surface map to show the infection density (cases per km^2^.) in the study area. In kernel density analysis, the search radius is regarded to have no impact on density calculation [25]. But it is different when the kernel density analysis map is used as an outbreak clustering map. As rabies transmits among dogs and from infected dogs to humans, for the purpose of the analysis, we assumed a search radius for the Gaussian kernel density analysis to reflect the accumulated range of infected dog movements before they bite and infect people [10].

### Average Nearest-neighbor Distance

Nearest neighbor distance method, initially introduced by J.G. Skellam (1952) and expanded by P.J. Clark and F.C. Evans (1954), is often used to test whether a set of disease cases is clustering [26], [27]. The observed distance is the average value of all nearest neighbor distances. The expected average distance is calculated based on a hypothetical random distribution of the same number of cases within the same area. The ANN (average nearest neighbor) ratio is calculated by dividing the observed average distance by the expected average distance. If the ANN ratio is less than 1, the case pattern is clustered. Otherwise, it is dispersed. Z-score is another factor to quantify the clustering degree. A higher absolute value of z-score indicates a higher degree of clustering. To compare the annual variation of the human rabies case distribution patterns, the case data were analyzed annually according to their time of infection.

### ST-DBSCAN

DBSCAN (Density-Based Spatial Clustering of Applications with Noise) [28] finds a number of clusters starting from the estimated density distribution of corresponding nodes. A case *q* is *directly density-reachable* from a case *p* if their distance is less than a given distance*ε*; a case *q* is *density-reachable* from a case *p* if there is a sequence *p_1_,…p_n_* of cases with *p_1_* = *p* and *p_n_* = *q* where each *p_i+1_* is *directly density-reachable* from *p_i_*. Compared to DBSCAN in which only spatial distance is used, we have extended the spatial distance to composite a spatiotemporal distance and defined it as: D_st_ = D_s_+δ*D_t_, where *D_s_* is spatial distance, *D_t_* is temporal distance, and *δ* is a ratio of temporal to spatial. We used Ordering Points To Identify the Clustering Structure (OPTICS) [29] to detect the density-based cluster distribution then to determine the parameters.

### Estimation of Geographical Spread

To analyze the spatial variation in the contiguous spatiotemporal variation of rabies spread and possible recovery, we made two continuous surface maps named first-appearance-map and last-appearance-map, respectively. A regular grid of discrete points was created covering the whole study area. In the first-appearance-map, each point was assigned with the date of the first reported human rabies case within a specific search radius. Then we used Inverse Distance Weight (IDW) interpolation to produce a contiguous surface map to sketch the annual spread front. Similarly, the last-appearance-map, which reflected the date of the last appearance of human rabies and its recovery surface map, was produced to discover the spatiotemporal recovery process. Previously infected areas where no new human rabies cases were reported for more than five years were regarded as recovered areas.

## Results

### Annual Spatial Distribution and the Density of Human Rabies Cases

**Figure 1:**
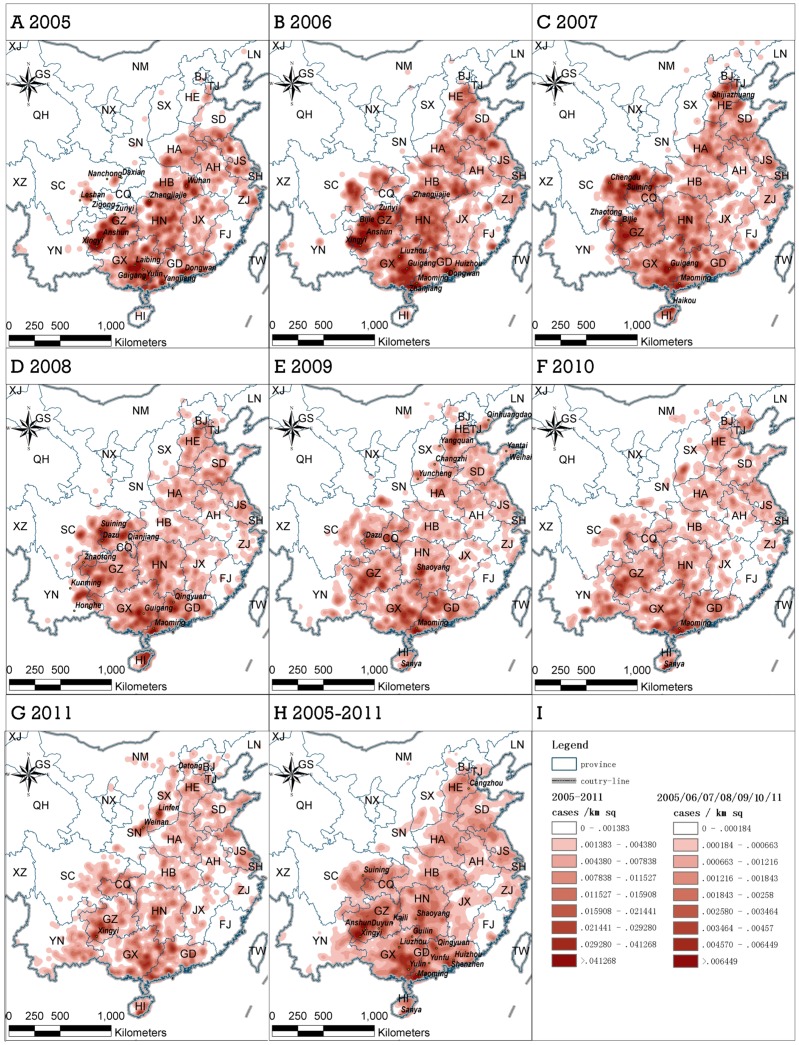
Gaussian kernel density surface maps of annual human rabies cases in mainland China from 2005 to 2011 (A-G) and the total cases during this period (H). (I) Legend of density rating. (The abbreviation of provinces: Anhui: AH, Beijing: BJ, Chongqing: CQ, Fujian: FJ, Gansu: GS, Guangdong: GD, Guangxi: GX, Guizhou: GZ, Hainan: HI, Hebei: HE, Heilongjiang: HL, Henan: HA, Hubei: HB, Hunan: HN, Inner Mongolia: NM, Jiangsu: JS, Jiangxi: JX, Jilin: JL, Liaoning: LN, Ningxia: NX, Qinghai: QH, Shaanxi: SN, Shandong: SD, Shanghai: SH, Shanxi: SX, Sichuan: SC, Taiwan: TW, Tianjing: TJ, Tibet: XZ, Xinjiang: XJ, Yunnan: YN, Zhejiang: ZJ).

We carried out a sensitivity analysis on the choice of search radius in Gaussian kernel density by comparing the results for search radius of 15, 25, 35, 50 and 75 kilometers. Then we reported the results using a search radius of 50 kilometers as the empirical value. From 2005 to 2007 there was an increase in the number of reported rabies cases followed by a decline during 2008 to 2011 (Table 1). While the temporal variation in the numbers of infected townships was similar to the variation in the number of rabies cases, the number of counties with human rabies infection was relatively steady through the whole period. The rabies outbreak areas, especially those with the highest infected areas varied spatially from 2005 to 2011(Figure 1) (Table 2).

**Table 1:** Distance (eucledian) to the neareast case of annual rabies data and clustered dataset.

Data set	Cases	Infected counties	Infected townships	Observed Mean Distance(kilometers)	Nearest Neighbor Ratio	z-score	Number of clusters detected by ST-DBSCAN
2005 Year	2537	710	2012	9.674	0.305	−67.114	88
2006 Year	3279	839	2568	10.565	0.474	−57.635	135
2007 Year	3300	984	2721	11.442	0.546	−49.897	106
2008 Year	2466	858	2060	14.596	0.409	−56.134	47
2009 Year	2213	892	1900	14.321	0.633	−33.053	33
2010 Year	2048	817	1750	15.339	0.578	−36.546	40
2011 Year	1917	862	1685	16.316	0.449	−46.142	31
Clustered cases 2005–2011 Year				4.823	0.306	−80.343	Sum:480

**Table 2:** Main spatial character and the highest infection areas of annual data.

Year	Pattern	The highest infected areas
2005	Only sporadic cases being reported in Shanxi, Shaanxi, and Sichuang	Wuhan of Hubei, Zhangjiajie of Hunan, Zunyi, Kaili, Anshun and Xingyi of Guizhou, border of Guigang, Laibing and Nanning of Guangxi, Yulin of Guangxi, Yangjiang and Dongguan of Guangdong
2006	The infection areas of Sichuan expanded and the density becamehigher. Nearly half of Shandong was infected	Zhangjiajie of Hunan, Zunyi and the borders between Anshun, Xingyi and Bijie of Guizhou, Guigang and Liuzhou of Guangxi, Maoming, Zhanjiang, and the crossing areas among Dongguan, Shenzhen and Huizhou of Guangdong.
2007	The infected areas in Sichuan continued to expand, covering thewhole region of Chongqing and reached the border betweenGuizhou and Hunan. The aggregated infection areas in Yunnan firstappeared in the border with Guizhou and Chongqing.	Shijiazhuang of Hebei, Chengdu, Suining, Nanchong and Guang’an of Sichuan, Bijie and Anshun of Guizhou, Guigang of Guangxi, Maoming of Guangdong, and Haikou of Hainan
2008	Both the number of cases and the size of serious infected areas were decreasing. More sporadic cases were reported	Suining and Dazu of Sichuan, Qianjiang of Hubei, Guigang of Guangxi, and Maoming and Qingyuan of Guangdong
2009	The rabies cases were scattered in national scale, Shaanxi reportedaggregated cases in districts near Sichuan. Cases in Shanxi formedthree aggregation regions. Rabies covered Qinhuangdao; Yantai andWeihai.	Shaoyang of Hunan, Maoming of Guangdong, and Sanya of Hainan
2010	The total number of cases decreased, but cases became moreconcentrated. In Yunnan, the two heavily infected areas in theprevious year kept expanding and finally merged. The infectionareas in Shanxi and Shaanxi expanded slowly but continuously.	Maoming of Guangdong and Sanya of Hainan
2011	The overall situation of 2011 was similar to that of 2010.The infection areas in South of Yunnan, Shaanxi and Shanxi wasincreasing	Xingyi of Guizhou and Linfen of Shanxi
Accumulative situation 2005–2011		Anshun and Xingyi in Southwest and South of Guizhou and Maoming of Guangdong (the highest density regions); Yongzhou of Hunan, Yulin and Liuzhou of Guangxi, and Maoming of Guangdong (the secondary heavily infected regions).

### Clustering Degree of Annual Case Data

From 2005 to 2011, as shown in Table 1, the annual observed distance of nearest neighbors increased from 9.674 kilometers to 16.316 kilometers. The ANN ratios were greater than 0.30, with the maximum value 0.633 in 2009. The overall trend of z-score abstraction was increasing, though it decreased a little in 2010 and 2011. This means the clustering degree of human rabies was decreasing.

**Figure 2:**
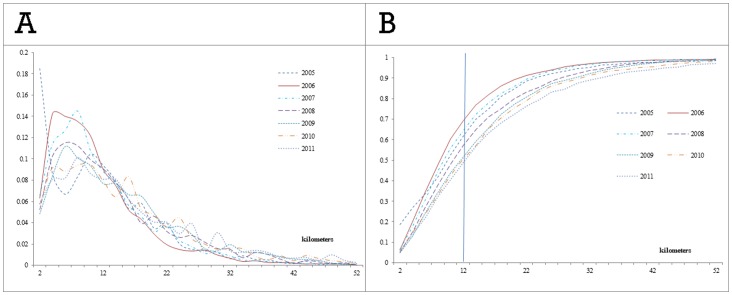
The distribution of nearest neighbor distances among human rabies cases (A) the proportion in terms of distance interval. (B) the accumulative proportion of nearest neighbor distances.

Most of nearest neighbor distances were distributed in the smaller distance range; and the proportion decreased with increasing distances (Figure 2-a). Overall, most nearest neighbors fell into the range of 6 to 10 kilometers. Cumulatively, nearly half of cases (the seven annual proportions of 62.0%, 69.3%, 64.7%, 57.2%, 51.5%, 50.6% and 48.9% respectively) had at least one neighbor in the range of 12 kilometer (Figure 2-b).

### Spatiotemporal Clustering

**Figure 3:**
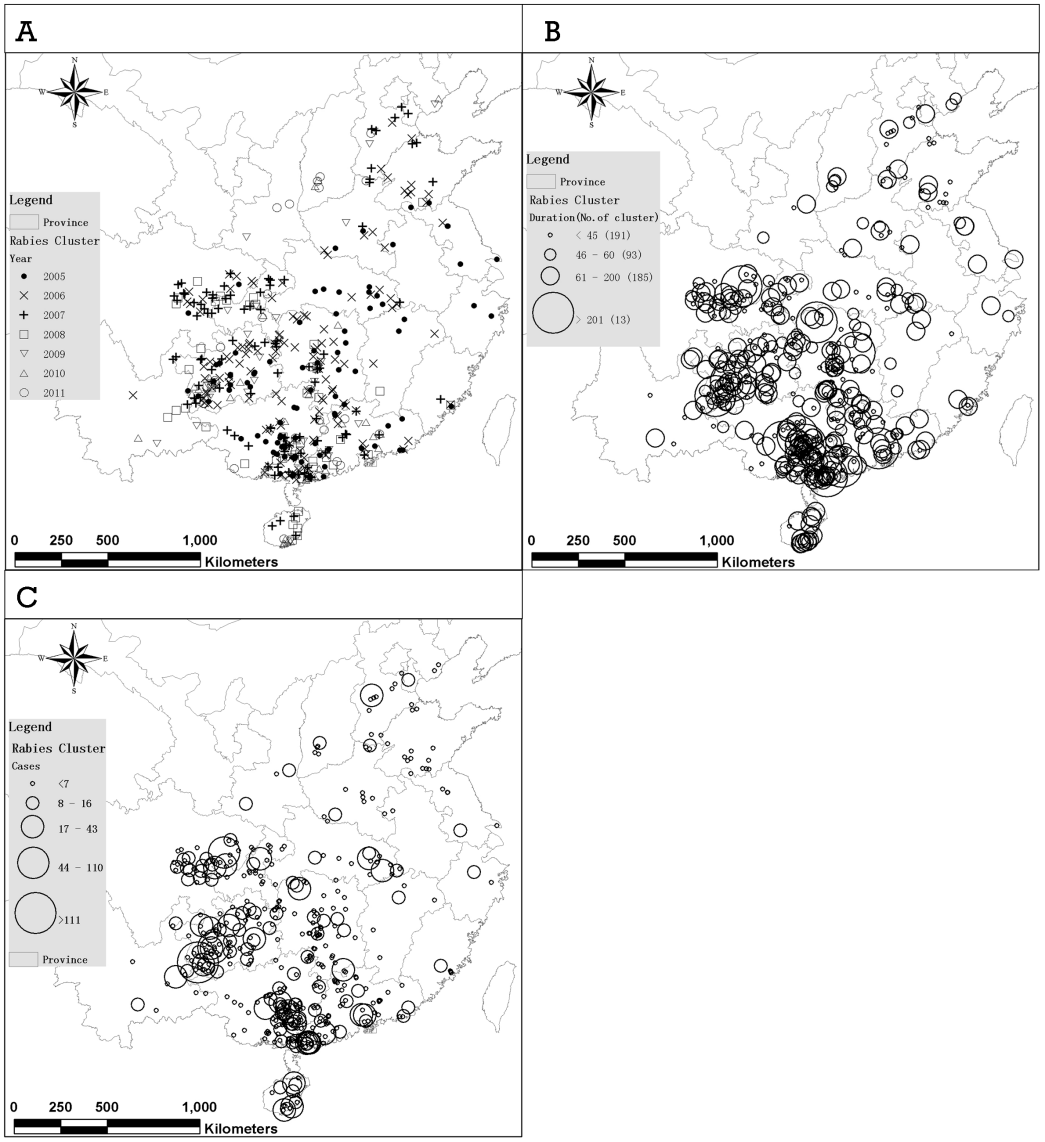
Spatial distribution of clusters detected by ST-DBSCAN by (A) occurrence time, (B) duration, and (C) the number of cases in clusters.

**Figure 4:**
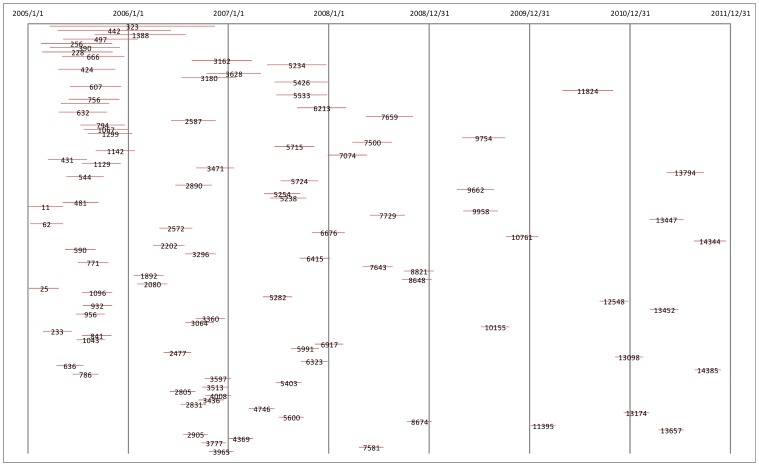
The Top 100 clusters detected by ST-DBSCAN in terms of their duration.

We compared the results calculated based on different spatio-temporal distances*ε_m_* = 10, 20, 30, 40, 50 kilometers and *δ = *0.25, 0.5, 1, 2 kilometers/day, and then chose the empirical value*ε_m_* = 30 kilometers, *δ = *0.5 kilometers/day, and the minimum point number *minPts* = 3. A total 480 clusters were detected by ST-DBSCAN, with an average duration of 39.73 days. The mean observed distance of ANN of clustered cases was 4.823 kilometer, and z-score was −80.343 (Table 1). The clusters that occurred after 2009 distributed mainly in North China Plain, Guizhou, Guangxi, Guangdong, Hainan, Sichuan and Yunnan. Those cluster, which lasted longer (>200 days) or with more cases (>110 cases), were mainly found in the Guizhou, Guangxi, Guangdong, Hainan, Sichuan and Yunnan (Figure 3). Those clusters which lasted longer than 200 days only appeared before July, 2007. Most of clusters initiated in the first three years (the proportion is 89.4%). A small number of clusters lasted years especially before 2009 (Figure 4).

### Spatiotemporal Spread and Recovery of Human Rabies

**Figure 5:**
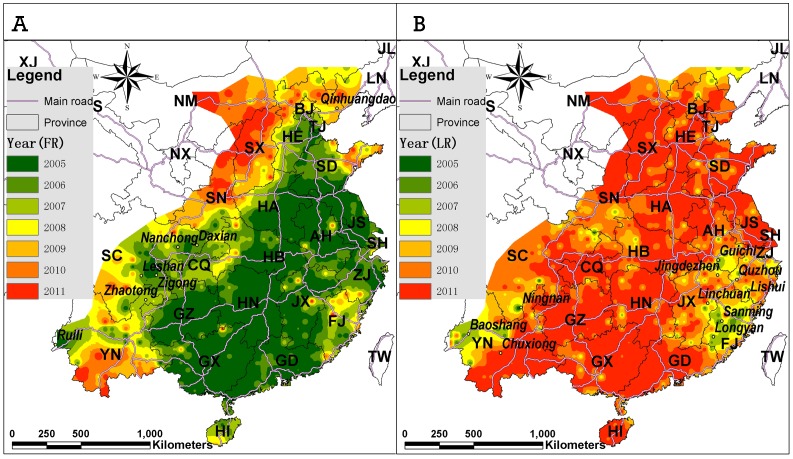
The spread and recover of human rabies. (A) The time of the first appearance of human rabies to identify the spread of human rabies, (B) The time of the last appearance of human rabies to detect the recovered areas of human rabies.

We categorized the study area into four regions to describe the geographic spread of human rabies according to variant spread patterns shown in Figure 1 and Figure 5-a. The first region was North of China, with well-developed transportation networks. From 2005 to 2007, the areas with concentrated cases kept expanding and the diffusion front reached the edge of Taihang Mountain to the west, the coastline of the Yellow Sea to the east, and Beijing, Tianjin, Inner Mongolia and East of Hebei to the north. Human rabies was reported in Shaanxi and Shanxi in 2009; and the infected areas of Shanxi and Shaanxi formed a contiguous infection region by 2010. The diffusion pattern in this region showed a distinct directional character, i.e. the rabies spread along the road network.

The second region consisted of Southwest of Sichuan, Chongqing, North of Guizhou, West of Hubei, and parts of Hunan. Before 2007, three separate infection areas were formed in Zigong, Neijiang and Leshan, and the border between Daxian and Nanchong of Sichuan. Then they merged into a large and contiguous region.

In Yunnan, only sporadic cases were reported in Ruili before 2007. After that, the two rabies aggregated areas, which respectively initiated in Zhaotong and East of Yunnan, expanded along the road network and merged in 2010.

The fourth region was Hainan Island, where only sporadic human rabies cases were reported in 2005. In the following years, rabies spread into the South part of the island along the roads. After 2007, most of the island was infected except the mountain areas in Southwest of Hainan.

From the last-appearance-map of rabies, for example, after 2006, few human rabies cases were reported in places of Fujian [30]. A similar situation was observed in Jiangxi, Zhejiang and South of Anhui. (Figure 5-b).

## Discussion

This study used comprehensive spatial analysis methodology to describe the spatiotemporal variation of human rabies infections in China. Analysis of the annual disease density for the period 2005 to 2011 suggests that rabies in China was spatially clustered. By integrating a temporal parameter in a novel density-based cluster detection algorithm, we were able to detect the location and duration of spatiotemporal rabies clusters. We then described the disease spread situation in these years and identified the recovery areas (areas previously infected, which have not reported new rabies cases for more than five years) and areas where rabies remains a considerable public health problem.

Compared to previous area-based (aggregated) studies, our approach based on point-based (geostatistical) data analysis shows remarkable spatiotemporal variation in rabies infections in China that previous studies failed to convey [9], [16], [23], [31]. Our results show that previous aggregated spatiotemporal analysis has masked important small scale geographical variation in human rabies cases by overlooking the spatial correlations among cases. For example, three adjacent counties Nanzheng, Hantai and Chenggu of Shaanxi province reported five rabies cases in 2011 and our results show that they occurred in a small area with an east-west length <25 kilometers, and south-north length <15 kilometers. While the risk for this area had been underestimated by previous analyses, our geostatistical approach was able to detect this high-risk cluster. This information will be useful for the local CDC and government in that it will help inform targeted human rabies control procedures, such as promote community awareness and knowledge of dog bite prevention, first aid and management of animal bites, resourcing local public health units for PEP delivery to prevent further geographical spread and human mortality and responsible dog ownership.

The annual Gaussian kernel density surface maps presented in this study identify areas where rabies cases were concentrated. While, the sequential spatial variation in annual density of rabies cases reveals that rabies spread widely across a large geographical landscape, these maps provide China CDC and local government authorities a scientific basis to target current high risk areas. With that regard, our maps suggest that the South and East of China were the primary infection areas from 2005 to 2011, and Yunnan and Shanxi became new infection areas after 2009 (Figure 2).

We used the distances between the nearest neighbor human rabies cases to examine the degree of clustering and the spatiotemporal rabies transmission, which cannot be identified by an area-based analysis. Based on the results of annual ANN distance, we found an increase in the length of rabies transmission pathways. The cornerstone to limiting the spread and containment of human rabies in China is the definition of rabies control areas [32]. The control areas are composed of two buffer areas centered on the location of rabies cases, named infected areas (with a radius of 3 kilometers) and risk areas (with a radius of 5 kilometers excluding the infected areas). In the infected areas the local CDC and government will cull infected dogs and restrict dog movement, while to reduce rabies transmission, mandatory vaccination of dogs is enforced in both the infected areas and the risk areas. The choice of the radius for the different sections of the control areas represents a compromise between resources and effective disease control. Our results for the observed ANN distance of clustered cases shows that, to prevent the spread of rabies from one rabies cluster to another, the radius of the infected areas could be increased to 5 kilometers (Table 1). Similarly, based on our results of the distances of nearest neighbors over time (Figure 2), extending the radius of the control areas to 12 kilometers (7 kilometers excluding the infected areas) would have effectively limited the transmission pathway to half of the rabies cases observed during 2005 to 2011. This finding is also supported by the results of the average observed nearest neighbor distance (13.18 kilometers, Table 1).

Taking into consideration the temporal dimension in cluster detection of rabies cases has provided renewed insights on our understanding of human rabies outbreak patterns in China. Besides the spatial distance (considered in ANN distance calculation and density analysis), temporal distance should also be considered when analyzing correlations among cases. The risk will be underestimated if rabies occurred densely in a short time only, as the risk is averaged annually. The results of the ST-DBSCAN have important public health implications in that they allowed the identification of high-risk areas where spatiotemporal rabies clusters lasted for years. For example, the outbreak, which took place in Anshun of Guizhou province, involved more than 100 cases and lasted from April 2005 to June 2006. A larger rabies outbreak took place in Xingyi and Liupanshui of Guizhou province, where more than 200 people died between March 2005 and November 2006.

We described the temporal dynamics of human rabies infected areas by rebuilding the infection status from its initiation and expansion to recovery. The first-appearance map identified the annual diffusion front and spreading patterns of human rabies from 2005–2011, and will help the national CDC make overall targeted control countermeasures to prevent rabies diffusion. This would be particularly important in areas such as Qinhuangdao, Zhaotong, Inner Mongolia, Shanxi, Shaanxi and Yunnan which could constitute priority areas for controlling rabies in the coming years in China. We have shown that rabies control interventions implemented by the national CDC and local government in Sanming, Longyan of Fujian and areas in Jiangxi, Zhejiang and Anhui have been successful at controlling human rabies cases in those areas. The rabies control measures and experiences which have proved successful in those areas should be documented and extended to other areas in the country.

The results of this study should be interpreted in light of the studies’ limitations. Firstly, the data on human rabies is generated by passive surveillance which means that additional rabies cases may have been missed during the study period. Second, sustained mass vaccination of domestic dogs is the cornerstone of the prevention of human rabies transmitted by dogs and geographical differences in dog vaccination coverage are likely to have an effect on the spatial patterns of human rabies identified in this study. Unfortunately we did not have access to data on dog vaccination coverage nor on other measures that impact on the transmission and outcome of dog bites such as dog management, promotion of community awareness and knowledge of dog bite prevention, quality of first aid and management of animal bites, resourcing for PEP and responsible dog ownership.

## Conclusions

Human rabies in mainland China is geographically clustered and its spatial extent changed during 2005 to 2011. The information conveyed by our analysis can be used by public health authorities to plan the delivery of human rabies prevention and control interventions, and to monitor and evaluate the effectiveness of such measures. Further studies are needed to explore the role of the natural, social factors and public health interventions on the spatial distribution identified in our study.
